# In Sentinel Node-Positive Melanoma Patients, Does Omission of Completion Lymph Node Dissection Make More Intensive Follow-Up Necessary, and Does Adjuvant Systemic Therapy Permit Less Intensive Follow-Up?

**DOI:** 10.1245/s10434-021-10572-3

**Published:** 2021-08-07

**Authors:** John F. Thompson

**Affiliations:** 1 Melanoma Institute Australia, The University of Sydney, North Sydney, NSW Australia; 2 Faculty of Medicine and Health, The University of Sydney, Sydney, NSW Australia; 3 Department of Melanoma and Surgical Oncology, Royal Prince Alfred Hospital, Sydney, NSW Australia

After the technique of sentinel node (SN) biopsy for accurately staging patients with primary cutaneous melanoma was introduced by Morton et al. in 1992,^1^ it soon became routine practice worldwide. The standard treatment for those found to be SN-positive was a completion lymph node dissection (CLND), and pathological examination of CLND specimens provided additional staging information by indicating whether additional metastatic disease was present in non-SNs. Before 1992, “elective” lymph node dissection of the relevant lymph node field for patients with intermediate thickness or thick primary melanomas had been widely practiced. However, because less than 20% of patients with melanomas > 1 mm in Breslow thickness were found to have metastatic melanoma in the resected nodes,^2^ the great majority had an unnecessary operation and many suffered significant morbidity from the surgery.

The reliability of the SN biopsy technique in identifying patients with metastatic melanoma in regional lymph nodes having been established in a large, international multicenter study, the first Multicenter Selective Lymphadenectomy Trial (MSLT-I),^3^ the next logical step was to determine whether immediate CLND improved the outcome for patients found to be SN-positive. Two multicenter studies were initiated to address this question, the German Dermatologic Cooperative Oncology Group Selective Lymphadenectomy Trial (DeCOG-SLT) and the much larger Second Multicenter Selective Lymphadenectomy Trial (MSLT-II). When the results of these two studies were published in 2016 and 2017,^4^,^5^ the management of SN-positive patients worldwide changed with amazing rapidity, and it was not long before few SN-positive patients were being offered CLND, with ultrasound monitoring of regional node fields offered instead. The “real world” outcomes to date have aligned closely with the randomized trial findings.^6^

The basis for the change from what had previously been regarded as standard of care for SN-positive melanoma patients was the finding in both MSLT-II and DeCOG-SLT that CLND was not associated with a significant survival benefit for SN-positive patients. However, there was lingering concern by clinicians because the results of MSLT-I and multiple retrospective studies had clearly shown that around 20% of SN-positive patients had metastatic disease in non-SNs when CLND specimens were examined. It was therefore anticipated that a similar percentage of patients would eventually develop detectable and likely more advanced metastatic disease in the node field if CLND was not performed, unless nodal disease that would in previous times have been removed by CLND could be eliminated by some form of adjuvant therapy.

Serendipitously, around the time that the MSLT-II and DeCOG-SLT results were published, the early results became available of trials demonstrating the value in patients with resected Stage III and Stage IV melanoma of adjuvant systemic therapy with immune checkpoint inhibitors (such as pembrolizumab and nivolumab) and agents targeting the BRAF/MEK pathway (such as dabrafenib, vemurafenib, and trametinib). These therapies had already been shown to be remarkably effective in treating unresected metastatic melanoma, achieving long-term disease control in around 50% of patients with advanced disease, albeit with quite frequent and sometimes serious side effects. The results of adjuvant systemic therapy trials after resection of metastatic melanoma have shown an improvement in relapse-free survival (RFS), but their long-term effects on melanoma-specific and overall survival are not yet well documented.^7^ However, no studies to date have reported detailed outcomes after the administration of adjuvant systemic therapy in patients who have had microscopic disease in a SN resected, where residual nodal disease inevitably remains in unresected non-SNs in some patients, as discussed above.

In this issue of *Annals of Surgical Oncology*, Broman et al.^8^ report the results of a study in which 177 SN-positive patients did not have a CLND; 66 of them received modern adjuvant systemic therapy, while the remaining 111 did not. This was an observational study, with inevitable limitations imposed by its retrospective, single-institution nature, its small patient numbers, and a median follow-up time of only 24 months. However, the observations are important because information on this topic is urgently needed. A rather surprising finding was that SN-positive melanoma patients managed without CLND had similar recurrence patterns with or without adjuvant systemic treatment. The great majority of nodal recurrences occurred within the first 12 months and were detected by physical examination or regular node field ultrasound examination. The particular focus of the study by Broman et al. was to determine whether the intensity of follow-up influenced outcome, and to document the cost-effectiveness of more intensive versus less intensive follow-up schedules.

Optimal follow-up surveillance strategies for patients who have been treated for primary melanoma by wide excision with or without SN biopsy have not been determined and are somewhat controversial,^9^ but there are ongoing attempts to assemble evidence indicating the cost-effectiveness of various follow-up schedules, notably the MELFO studies.^10^,^11^ Although longer follow-up of these studies is required, results to date have shown that a less intensive follow-up schedule, with fewer clinic visits and less frequent routine imaging tests, did not adversely affect outcomes and substantially reduced costs for both patients and healthcare systems. Until now, there has been no information about the efficacy and cost of various follow-up surveillance strategies for melanoma patients who have had a positive SN but not a CLND, as in the experimental arms of both the MSLT-II and DeCOG-SLT studies. In the study conducted by Broman et al., patients received varying levels of follow-up intensity in this clinical scenario. Based on the key finding that most nodal recurrences (85%) occurred within the first year, with 85% of these detected by clinical examination or by ultrasound, it was concluded that increased surveillance intensity did not improve recurrence detection rates but substantially increased overall cost (from $4,240 to $24,838) and cost per recurrence detected (from $15,688 to $82,792). Adjuvant treatment did not appear to alter patterns of initial recurrence, suggesting that whether a patient receives adjuvant therapy should not be a determinant of follow-up intensity. Broman et al. reported that regular PET/CT scans did not significantly improve the rate of detection of metastatic disease in regional lymph node fields but did increase costs considerably.

It is important to note that, in both MSLT-II and DeCOG-SLT, patients considered likely to have a higher risk of recurrence, i.e., those with microsatellites, extranodal extension from the SN, or more than three positive SNs, were excluded or underrepresented. In a recent large study by the International High-Risk Melanoma Consortium,^12^ a propensity score-matched analysis was performed using data for 166 patients who had one or more of these high-risk features. After a median follow-up of 18.5 months, 49% had developed a recurrence, compared with only 26% of patients without high-risk features (*p* < 0.01). However, for well-matched cohorts of these high-risk patients treated with or without CLND, there were no significant differences in recurrence at any site (CLND 49%, surveillance 45%, *p* = 0.99), node field recurrence (CLND 6%, surveillance 14%, *p* = 0.20) or melanoma-specific mortality (CLND 14%, surveillance 12%, *p* = 0.86). Although node field recurrences were somewhat (but not statistically) more frequent in the high-risk patients who did not have a CLND, most recurrences were at distant sites, supporting the use of nodal surveillance for this group also, as for the patients who were included in MSLT-II and DeCOG SLT.

Like any valuable piece of research, the study reported by Broman et al. goes some way to answering several questions but raises new ones, and indicates ways in which they might be addressed. Accepting the limitations of their study, it does seem to indicate that very intensive (and costly) follow-up of SN-positive melanoma patients who have not had a CLND does not improve outcomes—at least in the short to medium term. It also indicates that less intensive follow-up of these patients if they receive adjuvant systemic therapy is not likely to be appropriate. As longer-term outcome data become available, further studies will clearly be required to examine the cost-effectiveness of intensive and less intensive follow-up surveillance strategies for these patients.
